# Sustained live poultry market surveillance contributes to early warnings for human infection with avian influenza viruses

**DOI:** 10.1038/emi.2016.75

**Published:** 2016-08-03

**Authors:** Shisong Fang, Tian Bai, Lei Yang, Xin Wang, Bo Peng, Hui Liu, Yijie Geng, Renli Zhang, Hanwu Ma, Wenfei Zhu, Dayan Wang, Jinquan Cheng, Yuelong Shu

**Affiliations:** 1 Major Infectious Disease Control Key Laboratory, Key Reference Laboratory of Pathogen and Biosafety, Shenzhen Center for Disease Control and Prevention, Shenzhen 518055, China; 2 National Institute for Viral Disease Control and Prevention, Collaborative Innovation Center for Diagnosis and Treatment of Infectious Diseases, Key Laboratory for Medical Virology, Chinese Center for Disease Control and Prevention, Beijing 102206, China

**Keywords:** early warning, HPAI H5N6 virus, human infection, live poultry market, reassortant, surveillance

## Abstract

Sporadic human infections with the highly pathogenic avian influenza (HPAI) A (H5N6) virus have been reported in different provinces in China since April 2014. From June 2015 to January 2016, routine live poultry market (LPM) surveillance was conducted in Shenzhen, Guangdong Province. H5N6 viruses were not detected until November 2015. The H5N6 virus-positive rate increased markedly beginning in December 2015, and viruses were detected in LPMs in all districts of the city. Coincidently, two human cases with histories of poultry exposure developed symptoms and were diagnosed as H5N6-positive in Shenzhen during late December 2015 and early January 2016. Similar viruses were identified in environmental samples collected in the LPMs and the patients. In contrast to previously reported H5N6 viruses, viruses with six internal genes derived from the H9N2 or H7N9 viruses were detected in the present study. The increased H5N6 virus-positive rate in the LPMs and the subsequent human infections demonstrated that sustained LPM surveillance for avian influenza viruses provides an early warning for human infections. Interventions, such as LPM closures, should be immediately implemented to reduce the risk of human infection with the H5N6 virus when the virus is widely detected during LPM surveillance.

## INTRODUCTION

Poultry infections with the novel reassortant and highly pathologic avian influenza (HPAI) A (H5N6) viruses that have acquired the neuraminidase gene from H6N6 viruses of ducks in southern China have been reported in China, Laos and Vietnam since 2013.^1, 2, 3, 4, 5, 6^ The H5N6 viruses were also detected in swan geese and mammals, including cats and swine, in 2014.^7, 8^ The H5N6 viruses isolated from poultry and swine were found to be highly pathogenic in chickens and mildly virulent in BALB/c mice.^5, 7^ Since the first H5N6 human case was identified in April 2014,^9^ nine human infections have been reported in China.^9, 10, 11, 12^ Most of the patients had poultry exposure histories prior to illness onset and developed severe respiratory disease.^10^

Exposure to live poultry markets (LPMs) increases the risk of human avian influenza virus infection,^4, 13, 14, 15^ and LPM closures effectively control poultry-to-human transmission.^16^ However, the current strategy for LPM closures is primarily based on retrospective investigations that followed the detection or reporting of human infections. To investigate the role of LPMs in the poultry-to-human transmission of avian influenza viruses, a sustained LPM surveillance program was established in Shenzhen. Two human cases were detected shortly after the identification of H5N6 viruses in LPMs in Shenzhen in December 2015 and January 2016, and the potential linkage between human infection and the viruses in the LPMs was analyzed. Our study verified that sustained LPM surveillance could be an effective system for providing early warning about human infections with the avian influenza virus.

## MATERIALS AND METHODS

### Surveillance of avian influenza viruses in LPMs

There are ten districts in Shenzhen. The activity of avian influenza virus is associated with the environmental temperature. For example, lower-temperature conditions are associated with an increased risk of the survival of avian influenza viruses that have been implicated in human infections in LPMs.^17^ Therefore, we first conducted baseline surveillance of avian influenza viruses in LPMs in the Nanshan (NS) and Longgang (LG) districts of Shenzhen city in each month from June to November of 2015. From December 2015 to January 2016, we extended the monthly surveillance to all ten districts in Shenzhen by including the other eight districts of Longhuaxin, Futian, Guangming, Luohu, Pingshan, Dapeng, Bao'an and Yantian. There were no restrictions on the numbers or locations of the LPMs surveilled in each district. During each monthly sampling of each district, 10–20 environmental samples were collected from the same or different LPMs during this study period. The environmental samples included poultry feces, drinking water, sewage and swabs from poultry cages, feathers and poultry processing tools. The samples were maintained in viral transport medium and transferred immediately at low temperature to the local Centers for Disease Control and Prevention.

### Investigation, reporting and data collection from the patients

A standardized surveillance and reporting form was used to collect the following epidemiological and clinical data: demographic characteristics; underlying medical conditions; recent exposure to poultry, swine or other animals; recent visits to LPMs; clinical signs and symptoms; antiviral treatments; clinical complications; and outcomes. As a public health response to the outbreak, the requirement for the written informed consent of the patients was waived according to the Chinese law. Active surveillance was performed by collecting environmental samples in the epidemiologically implicated LPMs of the patients.

### RNA extraction and real-time reverse transcriptase–PCR

Viral RNA was extracted from all collected samples using a QIAamp Viral RNA Mini Kit (Qiagen, Hilden, Germany) according to the manufacturer's instructions. Specific real-time reverse transcriptase–PCR (RT-PCR) assay for FluA was initially used to screen all of the collected samples. Real-time RT-PCR assays for human seasonal influenza viruses (H1, H3 or B) were applied to the respiratory samples from the patients, and assays for the avian influenza H5, H7, H9, H10, N1, N6, N8 and N9 subtypes were performed for all samples to verify the viral subtypes.

### Virus isolation and genome sequencing

The samples that were detected as H5N6-positive were then subjected to virus isolation. The specimens were maintained in a viral transport medium and were inoculated and grown in nine- to ten-day-old specific pathogen-free (SPF) embryonated chicken eggs for 48-72 h at 37 °C. The full genomes of the virus isolates were amplified with a Qiagen OneStep RT-PCR Kit for sequencing (Qiagen, Germany).^18^ The PCR products were purified from an agarose gel with a QIAquick Gel Extraction Kit (Qiagen, Germany). Deep sequencing was performed at the Beijing Genomics Instituteten (BGI) using the Illumina HiSeq 2000 (Shenzhen, Beijing, China) platform. Sequences from the two human H5N6 viruses, that is, A/Shenzhen/1/2015 (SZ15) and A/Shenzhen/1/2016 (SZ16), were obtained from original respiratory samples from patients 1 and 2, respectively. Sequences of three environmental isolates, that is, A/Environment/Shenzhen/1/2015 (H5N6, E1) (Env1), A/Environment/Shenzhen/2/2015 (H5N6, E1) (Env2) and A/Environment/Shenzhen/3/2015 (H5N6, E1) (Env3), were obtained from passage viruses. The accession numbers of the viral sequences obtained in this study are provided in Supplementary Table S1.

### Genetic analysis

The identity of each viral gene was determined using MegAlign7.1. The data sets were obtained from the Global Initiative on Sharing All Influenza Data (GISAID) EpiFlu Database (Supplementary Table S2). Maximum likelihood phylogenetic trees of the nucleotide sequences of each of the genes of the selected influenza viruses were constructed with RAxML version 8.^19^ The bootstrap method with up to 1000 repetitions was used to test the phylogenies.

## RESULTS

### Increased H5N6 virus-positive rates of the LPMs associated with human H5N6 infection

**Figure 1:**
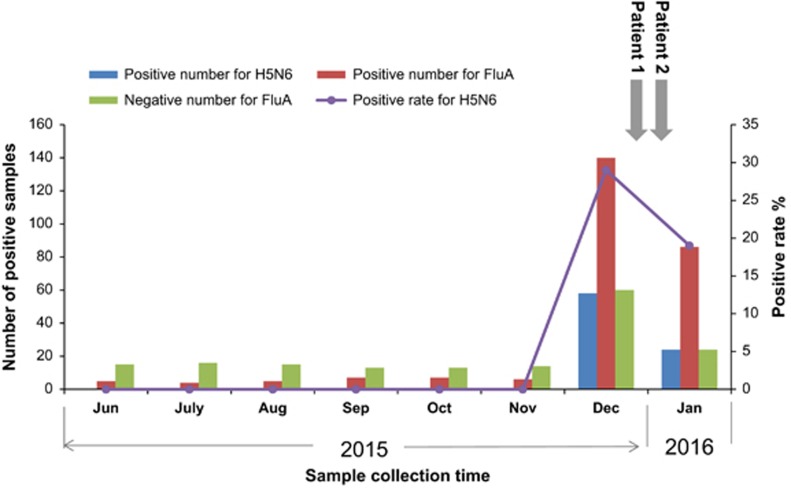
Results of routine surveillance of avian influenza viruses in LPMs in ten districts in Shenzhen. The RT-PCR positive numbers for FluA, H5N6 samples and negative numbers for FluA from June 2015 to January 2016 in the LPMs were showed in different colored columns. The H5H6-positive rates (%) during this study period were depicted using a purple curve in this figure. The gray arrows indicate the times of the illness onsets in patients 1 and 2. live poultry market, LPM; reverse transcriptase–PCR, RT-PCR.

From June 2015 to January 2016, routine surveillance of avian influenza viruses in the LPMs was conducted in the LG and NS districts of Shenzhen. Intensive LPM surveillance was conducted by collecting environmental samples from all 10 districts from December 2015 to January 2016. Before December, a total of 120 environmental samples were collected in selected LPMs in LG and NS districts. Positive samples were only detected in LG district. There were 34 out of 60 samples positive for FluA, but the avian influenza virus subtypes H5N6, H7N9 and H10N8 were not detected, with the exception of the H9N2 virus, which exhibited a positive rate of 25% (95% confidence interval (CI) 20%–30% Supplementary Table S3). The virus was continually detected in all 10 districts beginning in December 2015 (December: 34.5%, 95% CI: 21%–48%, January: 53, 95% CI: 33%–73%) but the subtype H7N9 and H10N8 viruses were still not detected in any of the LPMs in our study area. Notably, considerable increases in the H5N6 RT-PCR-positive rate (December: 29%, 95% CI: 14%–44%, January: 19%, 95% CI: 3%–35%) were observed beginning in December 2015 in all 10 districts (Supplementary Table S3 and Figure 1), which suggested that the H5N6 virus had circulated widely in LPMs in Shenzhen and posed a high risk for potential human infection during this period. Meanwhile, two human H5N6 virus infection cases were detected between late December and early January in Shenzhen, Guangdong Province, during the period of the increased H5N6 virus-positive rates in the LPMs (Figure 1). We proposed that sustained surveillance in LPMs may show some evidence that the risk of human infection might increase along with the increased activity of H5N6 virus in LPMs. The incidence of two new cases infected with H5N6 virus might be avoidable assuming that some interventions such as LPMs closures were implemented when the virus were widely detected via our surveillance.

### Epidemiological investigation of the patients confirmed that LPM exposure was potentially the cause of the human infections

Patient 1 was a 26-year-old female without underlying diseases. She developed fever and cough on 24 December 2015 and deteriorated with a high fever (40.2 °C) on 26 December. The patient was hospitalized on 27 December and treated with mechanical ventilation. Tamiflu was immediately administered to the patient. The patient eventually died from severe pneumonia, respiratory failure and septic shock on the 30 December 2015. Respiratory samples were collected on 28 December and found to be HPAI H5N6-positive.

Patient 2 was a 25-year-old healthy man who runs a small restaurant in Shenzhen. On 1 January 2016, the patient developed a high fever (39.5 °C), cough and dyspnea. On 6 January, the patient was hospitalized and diagnosed with severe pneumonia, Type 1 respiratory failure, acute respiratory distress syndrome, left pleural effusion and an electrolyte disorder. He was immediately administered antiviral treatment with Tamiflu (150 mg bid). Respiratory samples were collected on the 6th of January and found to be HPAI H5N6-positive. Combined antiviral therapy with Tamiflu (150 mg bid), zanamivir (10 mg each time, bid) and peramivir (300 mg qd) was then administered on 7 January. The patient eventually recovered from his severe condition.

Epidemiological investigations of the infection sources of both human H5N6 infections were conducted when the patients were confirmed as H5N6-positive. Patient 1 bought a duck from a neighboring LPM approximately four days prior to the onset of her illness. Patient 2 reported a history of visiting an LPM prior to the onset of his illness. Environmental samples and swabs of poultry feathers collected from the epidemiologically identified LPMs tested positive for the H5N6 virus (Supplementary Table S4). This result indicated that the LPMs were the potential sources of the human infections.

### Genetics and molecular characterization revealed the LPMs as the human infection source

**Figure 2:**
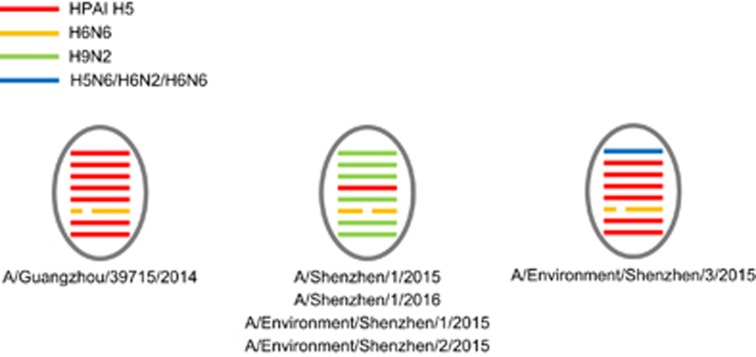
Genome comparisons of the H5N6 viruses in this study. The virus is indicated with a circle. The eight gene segments (horizontal bars) include, from top to bottom, PB2, PB1, PA, HA, NP, NA, MP and NS. The different lineages of the avian influenza viruses are differently colored. hemagglutinin, HA; matrix protein, MP; neuraminidase, NA; nucleoprotein, NP; non-structural protein, NS; polymerase acidic protein, PA; polymerase basic protein 1, PB1.

**Figure 3:**
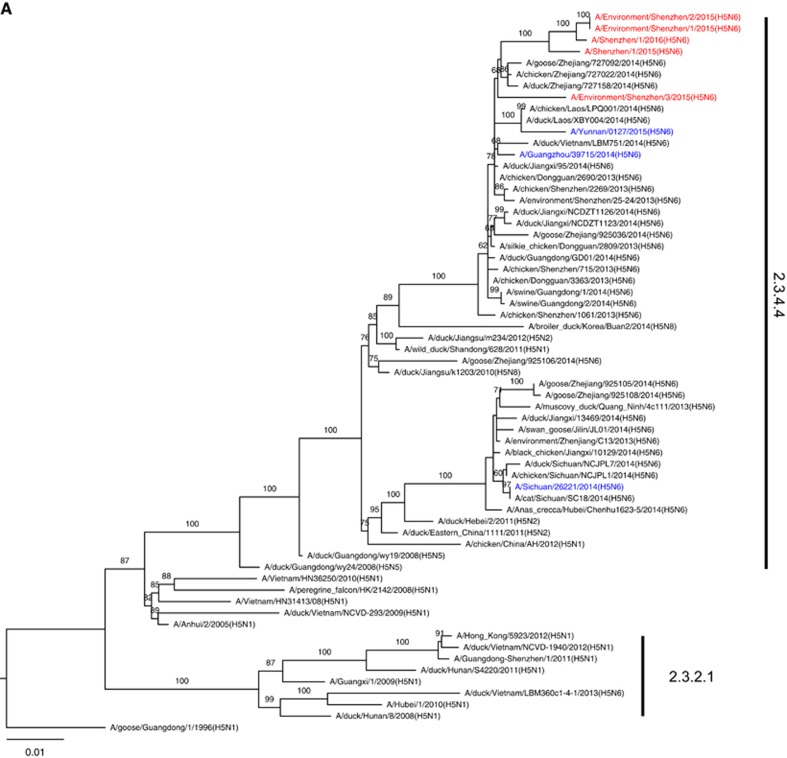

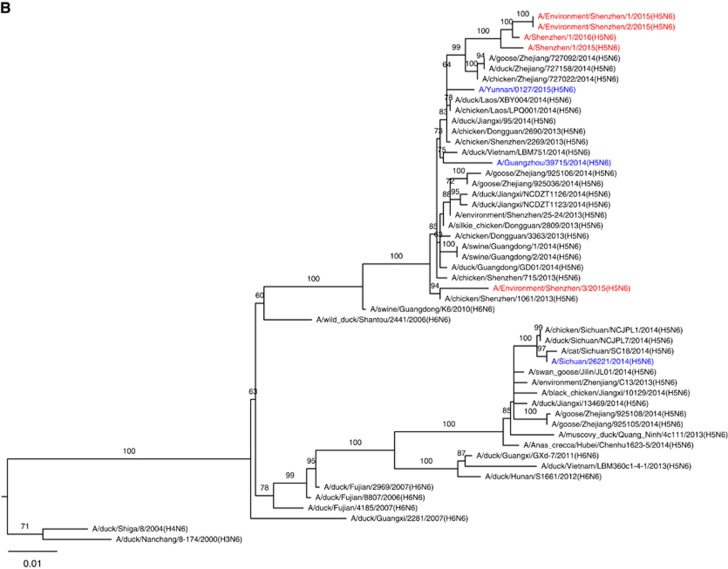
Phylogenic trees of the *HA* and *NA* genes. (**A**) Phylogenic trees of the *HA* gene. The virus names in blue represent previously reported human H5N6 viruses, and those in red represent the H5N6 viruses identified in this study. (**B**) Phylogenic trees of the *NA* gene. The virus names in blue represent previously reported human H5N6 viruses, and those in red represent the H5N6 viruses identified in this study. Bootstrap values ≥60% are shown. hemagglutinin, HA; neuraminidase, NA.

**Figure 4:**
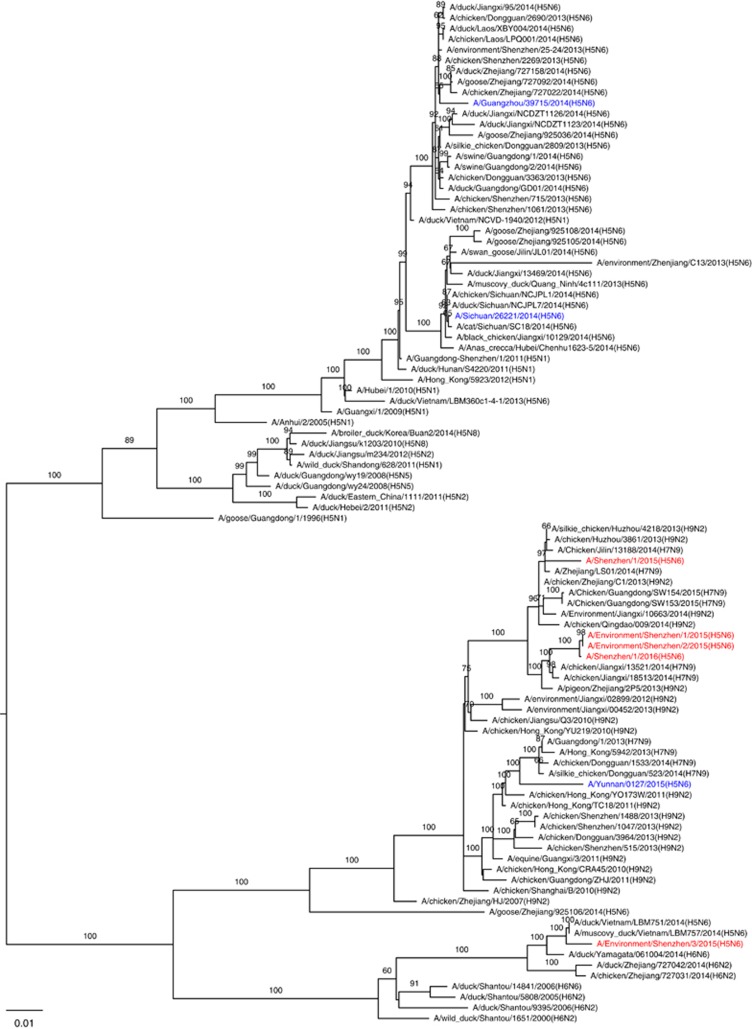
Phylogenic tree of the *PB2* gene. The virus names in blue represent previously reported human H5N6 viruses, and those in red represent the H5N6 viruses identified in this study. Bootstrap values ≥60% are shown. Polymerase basic protein 2, PB2.

The full genome sequences based on original respiratory samples from patients 1 and 2 were identified as A/Shenzhen/1/2015 (H5N6) (SZ15) and A/Shenzhen/1/2016 (H5N6) (SZ16), respectively. Three environmental viruses, that is, A/Environment/Shenzhen/1/2015 (H5N6) (Env1), A/Environment/Shenzhen/2/2015 (H5N6) (Env2) and A/Environment/Shenzhen/3/2015 (H5N6) (Env3), were isolated from environmental samples collected during our routine LPM surveillance in early December 2015. SZ16 shared the highest nucleotide identity (99.4-99.9%) with Env1 and Env2, and the identities between SZ15 and these two environmental isolates ranged from 96.7 to 99.3%. However, Env3 exhibited greater divergences with the other H5N6 viruses in this study (83.9–97.5%). Genetic and phylogenetic analyses revealed that SZ15 and SZ16 were reassortant viruses between the H5 of clade 2.3.4.4, H6N6 of Eurasian lineage and H9N2/H7N9 viruses (Figure 2). The hemagglutinin (*HA*) and neuraminidase genes of all five H5N6 viruses identified in Shenzhen were located in the same lineage of A/Guangzhou/39715/2014 (GZ39715, H5N6; Figure 3). However, all six internal genes of SZ15 and SZ16 clustered with the H9N2 or H7N9 viruses that were circulating in poultry, whereas the internal genes of GZ39715 belonged to H5N1 (Figure 4 and Supplementary Figure S1). The polymerase acidic protein (*PA*), polymerase basic protein 1 (*PB1*), nucleoprotein (*NP*), matrix protein (*MP*) and non-structural protein (*NS*) genes of Env3 were located in the H5 linage, which was similar to GZ39715 (Supplementary Figure S1). The *PB2* gene was located in a different sub-lineage with the H5N6, H6N6 and H6N2 viruses from birds (Figure 4). These results demonstrated the genetic diversity of the HPAI H5N6 viruses circulating in the LPMs. The high sequence identities of the viruses from the patients and the LPMs further confirmed the linkage between the human infections and LPM exposure. Multiple basic amino acids were present at the cleavage site between HA1 and HA2, which indicated that these viruses were highly pathogenic in chickens (Table 1). Mutations (S123P, I151T and T156A; H5 numbering) in HA related to an enhanced human-like receptor binding ability were detected in the human and environmental viruses (Table 1). The I368V mutation at PB1, which has been reported to be associated with increased transmissibility in ferrets, was detected in the present study. The amino acid lengths of PB1-F2 in the human and two of the environmental H5N6 isolates were longer than those of previously reported human H5N6 viruses, which indicated the pathogenicity of these viruses in mice. The C-terminal truncations of NS1 partially or completely removed its C-terminal tail, which contains features that might be involved in the biological activities of NS1. These features include the PDZ-binding motif, which is composed of NS1's four C-terminal residues and generally consists of the motif 227ESEV230 in the NS1 of avian influenza viruses.^20^ The avian C-terminal ESEV motif of NS1 increases virulence in mice,^21^ but truncations were detected in all of the isolates in this study. All isolates were resistant to adamantane derivatives but were susceptible to neuraminidase inhibitors. However, the PB2 E627K mutation that is associated with an increased adaptation and virulence in mammals was detected only in SZ16.

## DISCUSSION

LPM exposure is known to be an important source of the poultry-to-human transmission of avian influenza viruses, including the H5N1, H7N9 and H10N8 viruses.^14, 22, 23, 24^ To monitor the viral activities in LPMs in Shenzhen, a routine LPM surveillance system was established in 2013. In our study, no H5N6 virus was detected in the LPMs in Shenzhen prior to December 2015. However, markedly increased H5N6 virus frequencies in the LPMs were detected in all of the sampling sites distributed across the entire city beginning in December 2015. Two human cases of H5N6 virus infection were detected coincident with the increase in viral activity, and the epidemiological and genetic investigation results confirmed that the human infections were associated with LPM exposure. These results suggest that sustained surveillance of LPMs could serve as an effective early warning that could provide the opportunity to conduct interventions before the emergence of human infections with zoonotic avian influenza viruses.

LPMs act as sites for the mixing of avian influenza viruses and play important roles in viral reassortment and human infection.^25, 26, 27, 28, 29, 30^ In the present study, the H9N2 virus provided internal genes for H5N6 viruses that were also found in the in H7N9 and H10N8 viruses. These findings suggest that the H9N2 virus might be the key contributor to the genesis of novel virus genotypes in poultry.^31^ One possible reason could be the wide circulation of the H9N2 virus in LPMs, which is supported by the results of our surveillance that revealed increases in the H9N2 virus-positive rates that ranged from 25% (95% CI: 20%–30%) to 53% (95% CI: 33%–73%) from June 2015 to January 2016 (Supplementary Table S3). Another reason that H9N2 viruses might contribute to novel genotypes is the high genetic compatibility of H9N2 viruses observed in previous studies that have indicated that the internal genes of H9N2 viruses increase replication and transmissibility in animals.^32, 33^ Moreover, some mammalian adaptation mutations have also been detected in the internal genes of H9N2. In addition, mutations (S123P, I151T, and T156A) associated with enhanced human receptor binding ability were found in HA of the H5N6 viruses in this study. Thus, the H5N6 viruses presented here might be associated with a greater risk of human infection.

Similar to human infections with the H5N1 and H7N9 viruses, people infected with the highly pathogenic avian influenza A (H5N6) virus develop severe symptoms, including pneumonia and acute respiratory distress syndrome, and thus the H5N6 virus poses a threat to humans.^9, 12^ LPM closures have been effective in the control of human risks of H7N9 and H5N1 virus infections.^16, 34, 35^ The risk of human infection might be reduced if related interventions, such as LPM closures, are immediately implemented when increases in the frequencies of viruses are widely identified in LPMs. On the basis of our findings, we propose that the decision for LPM closures in Shenzhen city might need to be executed before December. Moreover, the supervision and information distribution by local public health facilities should be strengthened to prevent the unofficial trading of live poultry during LPM closure periods, which is often neglected in routine LPM management measures.

## Figures and Tables

**Table 1:** Characterizations of selected molecular markers of the H5N6 human viruses

**Gene**	**Phenotypic**	**Mutations**	**SZ16**	**SZ15**	**Env1**	**Env2**	**Env3**	**GZ/39715**	**SC/26221**
HA^a^	Altered receptor specificity	S123P	P	P	P	P	P	P	T
	Altered receptor specificity	I151T	T	T	T	T	T	T	I
	Altered receptor specificity, reduced transmission	T156A	A	A	A	A	A	T	A
	Highly pathogenic cleavage peptides		RERRRKR↓G	RERRRKR↓G	RERRRKR↓G	RERRRKR↓G	RERRRKR↓G	RERRRKR↓G	REKRRKR↓G
NA^b^		59–69 del	Yes	Yes	Yes	Yes	Yes	Yes	No
PB2	Altered virulence in mice	E627K	K	E	E	E	E	K	E
		D701N	D	D	D	D	D	D	N
PB1	Increased transmission in Ferret	I368V	V	V	V	V	I	I	I
PB1-F2	Increased pathogenicity in mice	87–90 AA in length	90 AA	90 AA	90 AA	90 AA	11 AA	11 AA	58 AA
PA	Species-associated signature positions	V100A	V	V	V	V	V	V	V
		K356R	R	R	R	R	K	K	K
		S409N	N	N	N	N	S	S	S
M2	Resistance to adamantane derivatives	S31N	N	N	N	N	S	S	S
NS1	Altered virulence in mice	80-84 del	No	No	No	No	Yes	Yes	Yes
		D92E	D	D	D	D	E	E	E
		L103F	L	L	L	L	F	F	F
		I106M	I	I	I	I	M	M	M
	Altered virulence in mice, PDZ motif	227-230	Truncated	Truncated	Truncated	Truncated	ESEV	ESEV	ESEV

Abbreviations: deleted, del; A/Environment/Shenzhen/1/2015, Env1; A/Environment/Shenzhen/2/2015, Env2; A/Environment/Shenzhen/3/2015, Env3; A/Guangzhou/39715/2015, GZ/39715; hemagglutinin, HA; neuraminidase, NA; nucleoprotein, NP; non-structural protein, NS; polymerase acidic protein, PA; polymerase basic protein 1, PB1; A/Shenzhen/1/2016, SZ16; A/Shenzhen/1/2015, SZ15; A/Sichuan/26221/2015, SC/26221.

a The H5 numbering system.

b The N6 numbering system.
