# Supplementary material for: Sustained live poultry market surveillance contributes to early warnings for human infection with avian influenza viruses
Source: Emerg Microbes Infect. 2016 Aug 3;5(8):e79–. doi: 10.1038/emi.2016.75 (PMC5034097; doi:10.1038/emi.2016.75)
Supplement: Supplementary Information [file emi201675x1.pdf]

a(PB1)

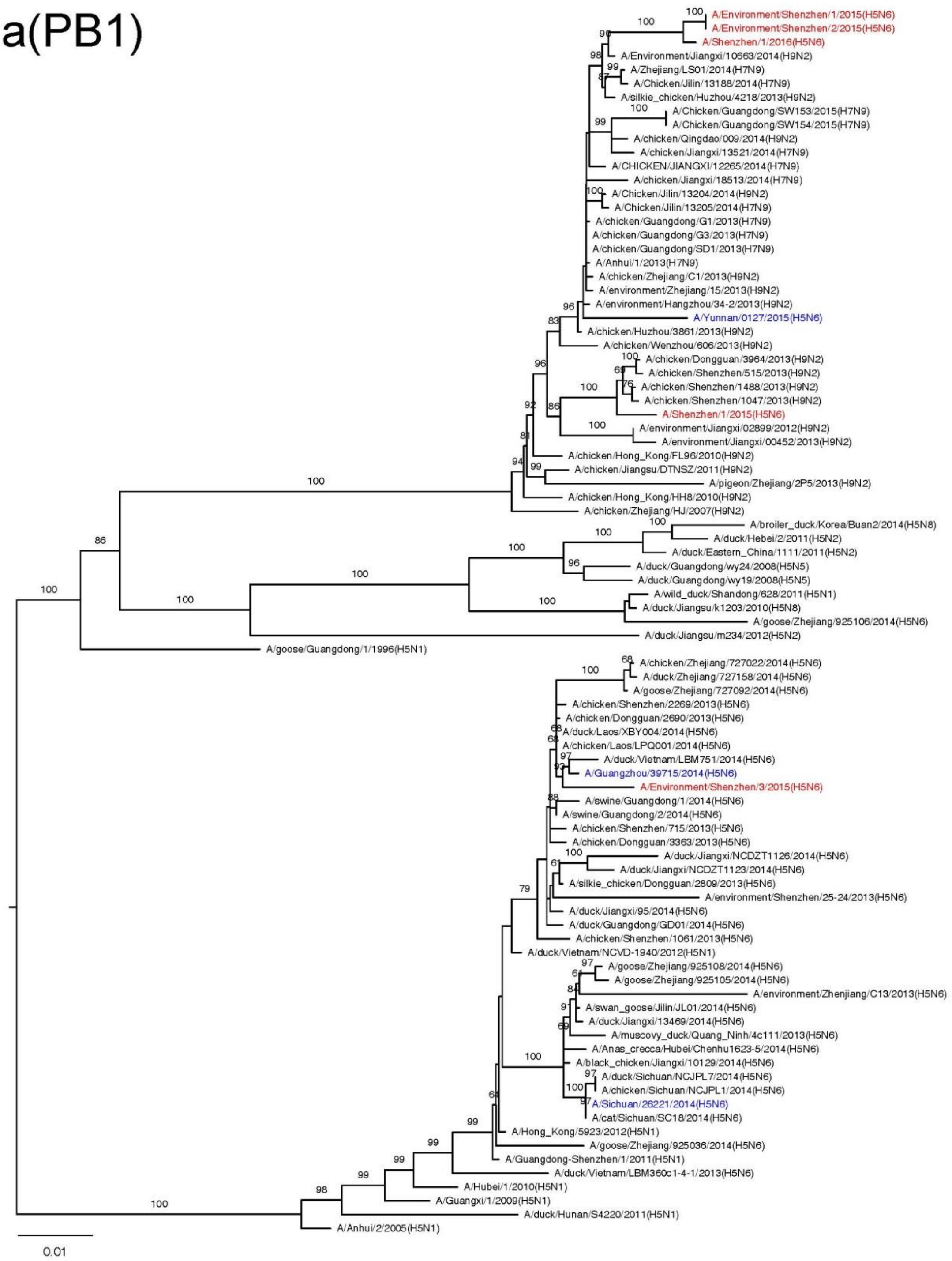
A/Environment/Shenzhen/1/2015(H5N6)
A/Environment/Shenzhen/2/2015(H5N6)
A/Shenzhen/1/2016(H5N6)
A/Environment/Jiangxi/10663/2014(H9N2)
A/Zhejiang/LS01/2014(H7N9)
A/Chicken/Jilin/13188/2014(H7N9)
A/silkie_chicken/Huzhou/4218/2013(H9N2)
A/Chicken/Guangdong/SW153/2015(H7N9)
A/Chicken/Guangdong/SW154/2015(H7N9)
A/chicken/Qingdao/009/2014(H9N2)
A/chicken/Jiangxi/13521/2014(H7N9)
A/CHICKEN/JIANGXI/12265/2014(H7N9)
A/chicken/Jiangxi/18513/2014(H7N9)
A/Chicken/Jilin/13204/2014(H9N2)
A/Chicken/Jilin/13205/2014(H7N9)
A/chicken/Guangdong/G1/2013(H7N9)
A/chicken/Guangdong/G3/2013(H7N9)
A/chicken/Guangdong/SD1/2013(H7N9)
A/Anhui/1/2013(H7N9)
A/chicken/Zhejiang/C1/2013(H9N2)
A/environment/Zhejiang/15/2013(H9N2)
A/environment/Hangzhou/34-2/2013(H9N2)
A/Yunnan/0127/2015(H5N6)
A/chicken/Huzhou/3861/2013(H9N2)
A/chicken/Wenzhou/606/2013(H9N2)
A/chicken/Dongguan/3964/2013(H9N2)
A/chicken/Shenzhen/515/2013(H9N2)
A/chicken/Shenzhen/1488/2013(H9N2)
A/chicken/Shenzhen/1047/2013(H9N2)
A/Shenzhen/1/2015(H5N6)
A/environment/Jiangxi/02899/2012(H9N2)
A/environment/Jiangxi/00452/2013(H9N2)
A/chicken/Hong_Kong/FL96/2010(H9N2)
A/chicken/Jiangsu/DTNSZ/2011(H9N2)
A/pigeon/Zhejiang/2P5/2013(H9N2)
A/chicken/Hong_Kong/HH8/2010(H9N2)
A/chicken/Zhejiang/HJ/2007(H9N2)
A/broiler_duck/Korea/Buan2/2014(H5N8)
A/duck/Hebei/2/2011(H5N2)
A/duck/Eastern_China/1111/2011(H5N2)
A/duck/Guangdong/wy24/2008(H5N5)
A/duck/Guangdong/wy19/2008(H5N5)
A/wild_duck/Shandong/628/2011(H5N1)
A/duck/Jiangsu/k1203/2010(H5N8)
A/goose/Zhejiang/925106/2014(H5N6)
A/duck/Jiangsu/m234/2012(H5N2)
A/goose/Guangdong/1/1996(H5N1)
A/chicken/Zhejiang/727022/2014(H5N6)
A/duck/Zhejiang/727158/2014(H5N6)
A/goose/Zhejiang/727092/2014(H5N6)
A/chicken/Shenzhen/2269/2013(H5N6)
A/chicken/Dongguan/2690/2013(H5N6)
A/duck/Laos/XBY004/2014(H5N6)
A/chicken/Laos/LPQ001/2014(H5N6)
A/duck/Vietnam/LBM751/2014(H5N6)
A/Guangzhou/39715/2014(H5N6)
A/Environment/Shenzhen/3/2015(H5N6)
A/swine/Guangdong/1/2014(H5N6)
A/swine/Guangdong/2/2014(H5N6)
A/chicken/Shenzhen/715/2013(H5N6)
A/chicken/Dongguan/3363/2013(H5N6)
A/duck/Jiangxi/NCDZT1126/2014(H5N6)
A/duck/Jiangxi/NCDZT1123/2014(H5N6)
A/silkie_chicken/Dongguan/2809/2013(H5N6)
A/environment/Shenzhen/25-24/2013(H5N6)
A/duck/Jiangxi/95/2014(H5N6)
A/duck/Guangdong/GD01/2014(H5N6)
A/chicken/Shenzhen/1061/2013(H5N6)
A/duck/Vietnam/NCVD-1940/2012(H5N1)
A/goose/Zhejiang/925108/2014(H5N6)
A/goose/Zhejiang/925105/2014(H5N6)
A/environment/Zhenjiang/C13/2013(H5N6)
A/swan_goose/Jilin/JL01/2014(H5N6)
A/duck/Jiangxi/13469/2014(H5N6)
A/muscovy_duck/Quang_Ninh/4c111/2013(H5N6)
A/Anas_crecca/Hubei/Chenhu1623-5/2014(H5N6)
A/black_chicken/Jiangxi/10129/2014(H5N6)
A/duck/Sichuan/NCJPL7/2014(H5N6)
A/chicken/Sichuan/NCJPL1/2014(H5N6)
A/Sichuan/26221/2014(H5N6)
A/cat/Sichuan/SC18/2014(H5N6)
A/Hong_Kong/5923/2012(H5N1)
A/goose/Zhejiang/925036/2014(H5N6)
A/Guangdong-Shenzhen/1/2011(H5N1)
A/duck/Vietnam/LBM360c1-4-1/2013(H5N6)
A/Hubei/1/2010(H5N1)
A/Guangxi/1/2009(H5N1)
A/duck/Hunan/S4220/2011(H5N1)
A/Anhui/2/2005(H5N1)
0.01

b(PA)

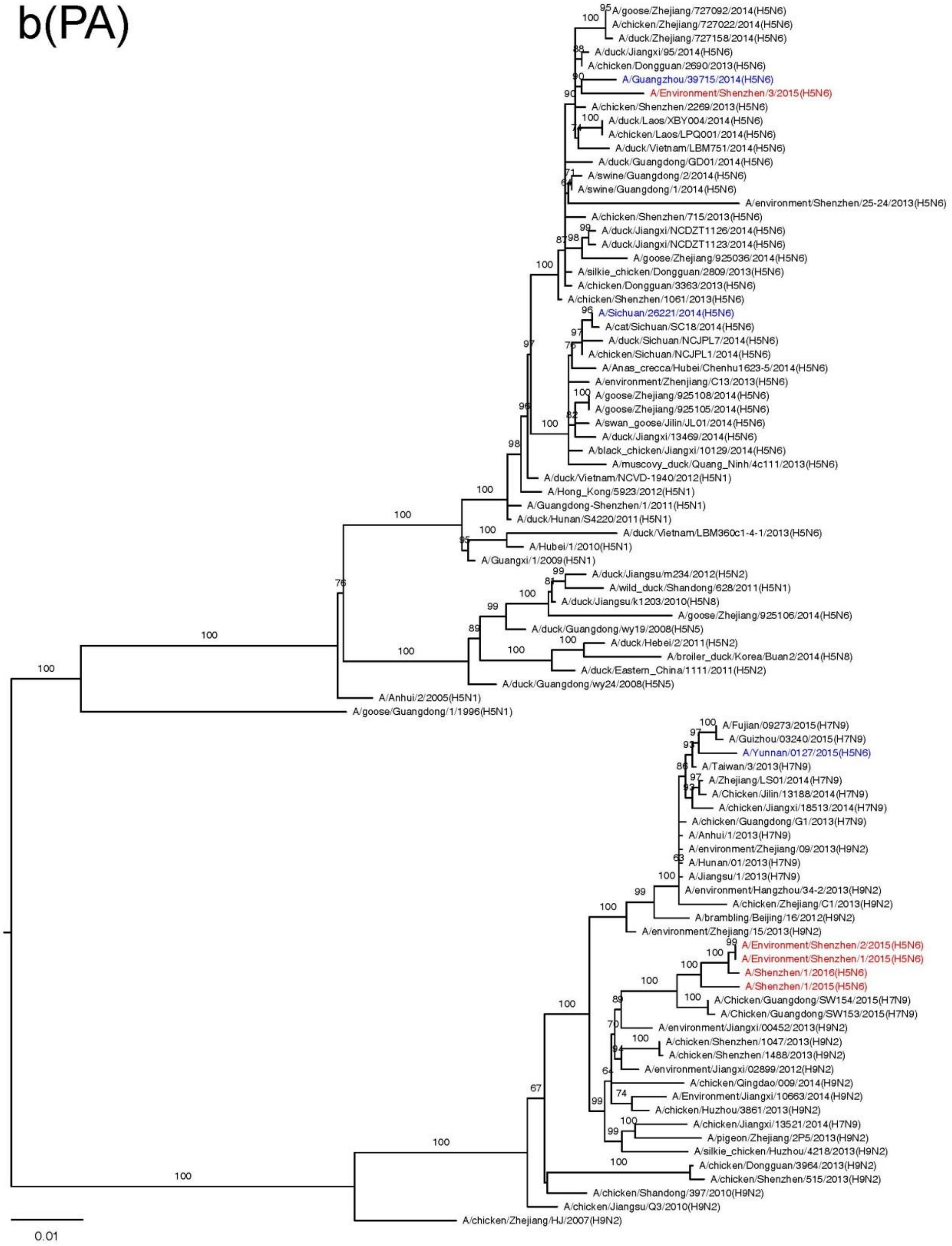
A/goose/Zhejiang/727092/2014(H5N6)
A/chicken/Zhejiang/727022/2014(H5N6)
A/duck/Zhejiang/727158/2014(H5N6)
A/duck/Jiangxi/95/2014(H5N6)
A/chicken/Dongguan/2690/2013(H5N6)
A/Guangzhou/39715/2014(H5N6)
A/Environment/Shenzhen/3/2015(H5N6)
A/chicken/Shenzhen/2269/2013(H5N6)
A/duck/Laos/XBY004/2014(H5N6)
A/chicken/Laos/LPQ001/2014(H5N6)
A/duck/Vietnam/LBM751/2014(H5N6)
A/duck/Guangdong/GD01/2014(H5N6)
A/swine/Guangdong/2/2014(H5N6)
A/swine/Guangdong/1/2014(H5N6)
A/environment/Shenzhen/25-24/2013(H5N6)
A/chicken/Shenzhen/715/2013(H5N6)
A/duck/Jiangxi/NCDZT1126/2014(H5N6)
A/duck/Jiangxi/NCDZT1123/2014(H5N6)
A/goose/Zhejiang/925036/2014(H5N6)
A/silkie_chicken/Dongguan/2809/2013(H5N6)
A/chicken/Dongguan/3363/2013(H5N6)
A/chicken/Shenzhen/1061/2013(H5N6)
A/Sichuan/26221/2014(H5N6)
A/cat/Sichuan/SC18/2014(H5N6)
A/duck/Sichuan/NCJPL7/2014(H5N6)
A/chicken/Sichuan/NCJPL1/2014(H5N6)
A/Anas_crecca/Hubei/Chenhu1623-5/2014(H5N6)
A/environment/Zhenjiang/C13/2013(H5N6)
A/goose/Zhejiang/925108/2014(H5N6)
A/goose/Zhejiang/925105/2014(H5N6)
A/swan_goose/Jilin/JL01/2014(H5N6)
A/duck/Jiangxi/13469/2014(H5N6)
A/black_chicken/Jiangxi/10129/2014(H5N6)
A/muscovy_duck/Quang_Ninh/4c111/2013(H5N6)
A/duck/Vietnam/NCVD-1940/2012(H5N1)
A/Hong_Kong/5923/2012(H5N1)
A/Guangdong-Shenzhen/1/2011(H5N1)
A/duck/Hunan/S4220/2011(H5N1)
A/duck/Vietnam/LBM360c1-4-1/2013(H5N6)
A/Hubei/1/2010(H5N1)
A/Guangxi/1/2009(H5N1)
A/duck/Jiangsu/m234/2012(H5N2)
A/wild_duck/Shandong/628/2011(H5N1)
A/duck/Jiangsu/k1203/2010(H5N8)
A/goose/Zhejiang/925106/2014(H5N6)
A/duck/Guangdong/wy19/2008(H5N5)
A/duck/Hebei/2/2011(H5N2)
A/broiler_duck/Korea/Buan2/2014(H5N8)
A/duck/Eastern_China/1111/2011(H5N2)
A/duck/Guangdong/wy24/2008(H5N5)
A/Anhui/2/2005(H5N1)
A/goose/Guangdong/1/1996(H5N1)
A/Fujian/09273/2015(H7N9)
A/Guizhou/03240/2015(H7N9)
A/Yunnan/0127/2015(H5N6)
A/Taiwan/3/2013(H7N9)
A/Zhejiang/LS01/2014(H7N9)
A/Chicken/Jilin/13188/2014(H7N9)
A/chicken/Jiangxi/18513/2014(H7N9)
A/chicken/Guangdong/G1/2013(H7N9)
A/Anhui/1/2013(H7N9)
A/environment/Zhejiang/09/2013(H9N2)
A/Hunan/01/2013(H7N9)
A/Jiangsu/1/2013(H7N9)
A/environment/Hangzhou/34-2/2013(H9N2)
A/chicken/Zhejiang/C1/2013(H9N2)
A/brambling/Beijing/16/2012(H9N2)
A/environment/Zhejiang/15/2013(H9N2)
A/Environment/Shenzhen/2/2015(H5N6)
A/Environment/Shenzhen/1/2015(H5N6)
A/Shenzhen/1/2016(H5N6)
A/Shenzhen/1/2015(H5N6)
A/Chicken/Guangdong/SW154/2015(H7N9)
A/Chicken/Guangdong/SW153/2015(H7N9)
A/environment/Jiangxi/00452/2013(H9N2)
A/chicken/Shenzhen/1047/2013(H9N2)
A/chicken/Shenzhen/1488/2013(H9N2)
A/environment/Jiangxi/02899/2012(H9N2)
A/chicken/Qingdao/009/2014(H9N2)
A/Environment/Jiangxi/10663/2014(H9N2)
A/chicken/Huzhou/3861/2013(H9N2)
A/chicken/Jiangxi/13521/2014(H7N9)
A/pigeon/Zhejiang/2P5/2013(H9N2)
A/silkie_chicken/Huzhou/4218/2013(H9N2)
A/chicken/Dongguan/3964/2013(H9N2)
A/chicken/Shenzhen/515/2013(H9N2)
A/chicken/Shandong/397/2010(H9N2)
A/chicken/Jiangsu/Q3/2010(H9N2)
A/chicken/Zhejiang/HJ/2007(H9N2)
0.01

c(NP)

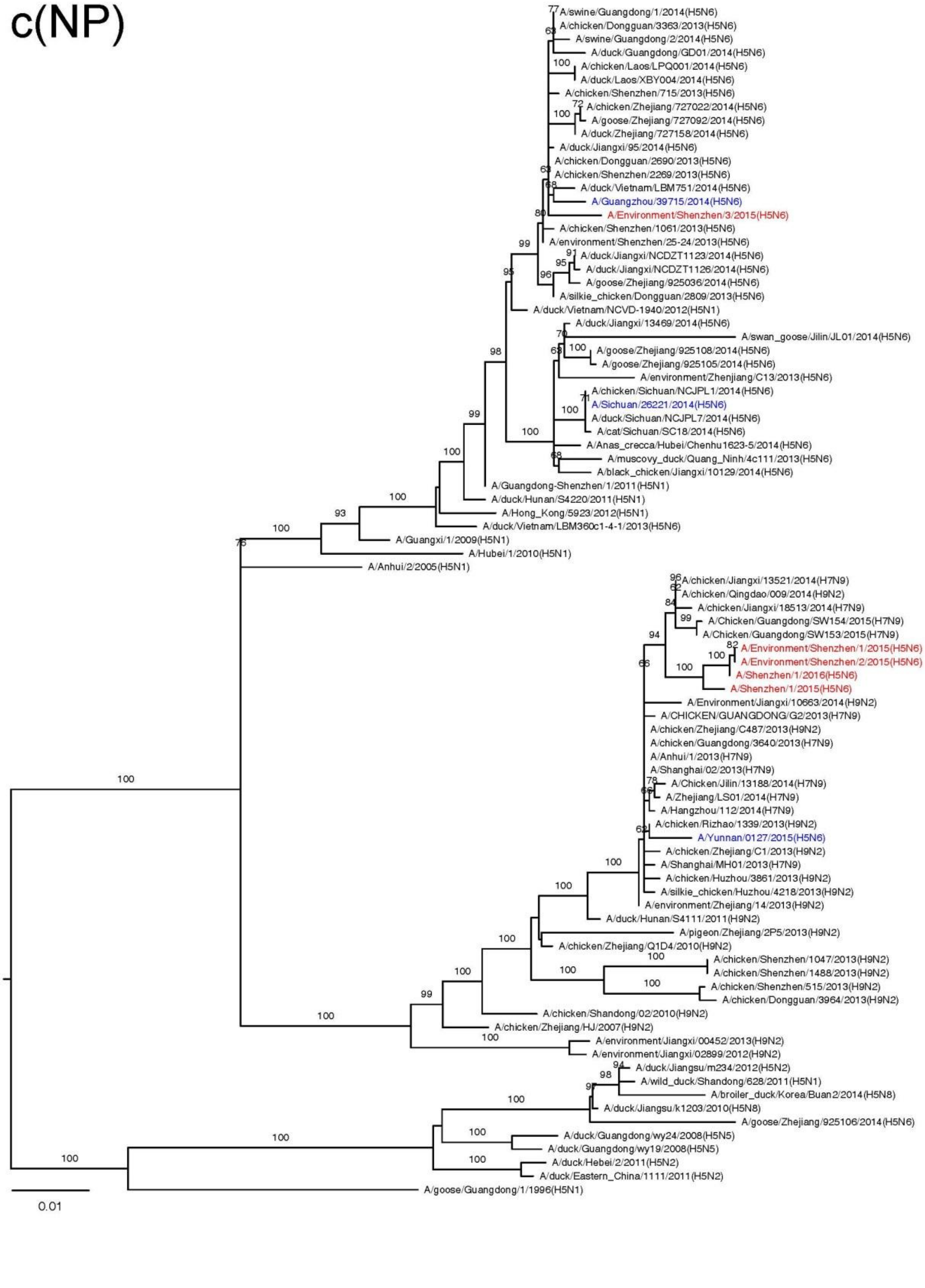
A/swine/Guangdong/1/2014(H5N6)
A/chicken/Dongguan/3363/2013(H5N6)
A/swine/Guangdong/2/2014(H5N6)
A/duck/Guangdong/GD01/2014(H5N6)
A/chicken/Laos/LPQ001/2014(H5N6)
A/duck/Laos/XBY004/2014(H5N6)
A/chicken/Shenzhen/715/2013(H5N6)
A/chicken/Zhejiang/727022/2014(H5N6)
A/goose/Zhejiang/727092/2014(H5N6)
A/duck/Zhejiang/727158/2014(H5N6)
A/duck/Jiangxi/95/2014(H5N6)
A/chicken/Dongguan/2690/2013(H5N6)
A/chicken/Shenzhen/2269/2013(H5N6)
A/duck/Vietnam/LBM751/2014(H5N6)
A/Guangzhou/39715/2014(H5N6)
A/Environment/Shenzhen/3/2015(H5N6)
A/chicken/Shenzhen/1061/2013(H5N6)
A/environment/Shenzhen/25-24/2013(H5N6)
A/duck/Jiangxi/NCDZT1123/2014(H5N6)
A/duck/Jiangxi/NCDZT1126/2014(H5N6)
A/goose/Zhejiang/925036/2014(H5N6)
A/silkie_chicken/Dongguan/2809/2013(H5N6)
A/duck/Vietnam/NCVD-1940/2012(H5N1)
A/duck/Jiangxi/13469/2014(H5N6)
A/swan_goose/Jilin/JL01/2014(H5N6)
A/goose/Zhejiang/925108/2014(H5N6)
A/goose/Zhejiang/925105/2014(H5N6)
A/environment/Zhenjiang/C13/2013(H5N6)
A/chicken/Sichuan/NCJPL1/2014(H5N6)
A/Sichuan/26221/2014(H5N6)
A/duck/Sichuan/NCJPL7/2014(H5N6)
A/cat/Sichuan/SC18/2014(H5N6)
A/Anas_crecca/Hubei/Chenhu1623-5/2014(H5N6)
A/muscovy_duck/Quang_Ninh/4c111/2013(H5N6)
A/black_chicken/Jiangxi/10129/2014(H5N6)
A/Guangdong-Shenzhen/1/2011(H5N1)
A/duck/Hunan/S4220/2011(H5N1)
A/Hong_Kong/5923/2012(H5N1)
A/duck/Vietnam/LBM360c1-4-1/2013(H5N6)
A/Guangxi/1/2009(H5N1)
A/Hubei/1/2010(H5N1)
A/Anhui/2/2005(H5N1)
A/chicken/Jiangxi/13521/2014(H7N9)
A/chicken/Qingdao/009/2014(H9N2)
A/chicken/Jiangxi/18513/2014(H7N9)
A/Chicken/Guangdong/SW154/2015(H7N9)
A/Chicken/Guangdong/SW153/2015(H7N9)
A/Environment/Shenzhen/1/2015(H5N6)
A/Environment/Shenzhen/2/2015(H5N6)
A/Shenzhen/1/2016(H5N6)
A/Shenzhen/1/2015(H5N6)
A/Environment/Jiangxi/10663/2014(H9N2)
A/CHICKEN/GUANGDONG/G2/2013(H7N9)
A/chicken/Zhejiang/C487/2013(H9N2)
A/chicken/Guangdong/3640/2013(H7N9)
A/Anhui/1/2013(H7N9)
A/Shanghai/02/2013(H7N9)
A/Chicken/Jilin/13188/2014(H7N9)
A/Zhejiang/LS01/2014(H7N9)
A/Hangzhou/112/2014(H7N9)
A/chicken/Rizhao/1339/2013(H9N2)
A/Yunnan/0127/2015(H5N6)
A/chicken/Zhejiang/C1/2013(H9N2)
A/Shanghai/MH01/2013(H7N9)
A/chicken/Huzhou/3861/2013(H9N2)
A/silkie_chicken/Huzhou/4218/2013(H9N2)
A/environment/Zhejiang/14/2013(H9N2)
A/duck/Hunan/S4111/2011(H9N2)
A/pigeon/Zhejiang/2P5/2013(H9N2)
A/chicken/Zhejiang/Q1D4/2010(H9N2)
A/chicken/Shenzhen/1047/2013(H9N2)
A/chicken/Shenzhen/1488/2013(H9N2)
A/chicken/Shenzhen/515/2013(H9N2)
A/chicken/Dongguan/3964/2013(H9N2)
A/chicken/Shandong/02/2010(H9N2)
A/chicken/Zhejiang/HJ/2007(H9N2)
A/environment/Jiangxi/00452/2013(H9N2)
A/environment/Jiangxi/02899/2012(H9N2)
A/duck/Jiangsu/m234/2012(H5N2)
A/wild_duck/Shandong/628/2011(H5N1)
A/broiler_duck/Korea/Buan2/2014(H5N8)
A/duck/Jiangsu/k1203/2010(H5N8)
A/goose/Zhejiang/925106/2014(H5N6)
A/duck/Guangdong/wy24/2008(H5N5)
A/duck/Guangdong/wy19/2008(H5N5)
A/duck/Hebei/2/2011(H5N2)
A/duck/Eastern_China/1111/2011(H5N2)
A/goose/Guangdong/1/1996(H5N1)
0.01

d(MP)

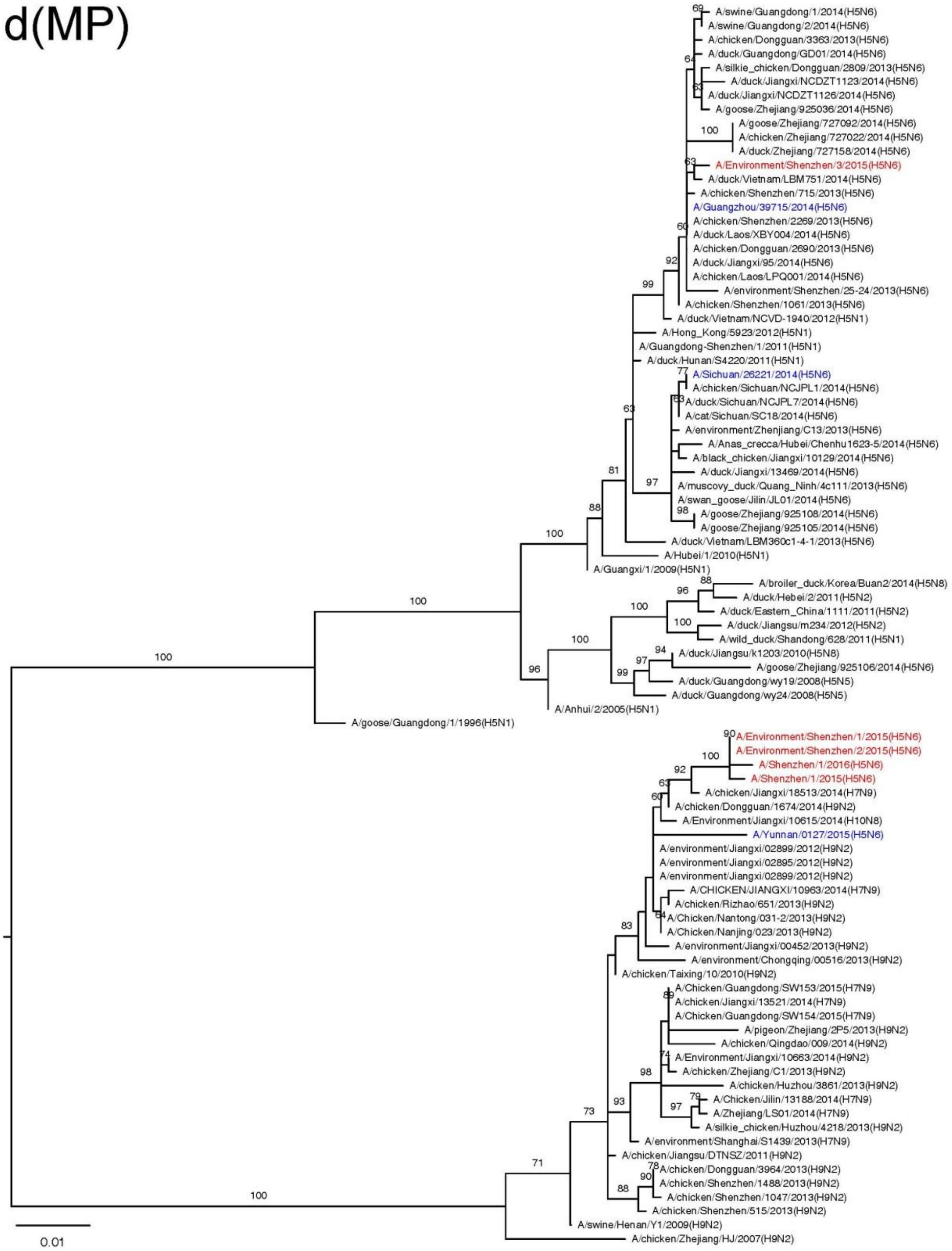
A/swine/Guangdong/1/2014(H5N6)
A/swine/Guangdong/2/2014(H5N6)
A/chicken/Dongguan/3363/2013(H5N6)
A/duck/Guangdong/GD01/2014(H5N6)
A/silkie_chicken/Dongguan/2809/2013(H5N6)
A/duck/Jiangxi/NCDZT1123/2014(H5N6)
A/duck/Jiangxi/NCDZT1126/2014(H5N6)
A/goose/Zhejiang/925036/2014(H5N6)
A/goose/Zhejiang/727092/2014(H5N6)
A/chicken/Zhejiang/727022/2014(H5N6)
A/duck/Zhejiang/727158/2014(H5N6)
A/Environment/Shenzhen/3/2015(H5N6)
A/duck/Vietnam/LBM751/2014(H5N6)
A/chicken/Shenzhen/715/2013(H5N6)
A/Guangzhou/39715/2014(H5N6)
A/chicken/Shenzhen/2269/2013(H5N6)
A/duck/Laos/XBY004/2014(H5N6)
A/chicken/Dongguan/2690/2013(H5N6)
A/duck/Jiangxi/95/2014(H5N6)
A/chicken/Laos/LPQ001/2014(H5N6)
A/environment/Shenzhen/25-24/2013(H5N6)
A/chicken/Shenzhen/1061/2013(H5N6)
A/duck/Vietnam/NCVD-1940/2012(H5N1)
A/Hong_Kong/5923/2012(H5N1)
A/Guangdong-Shenzhen/1/2011(H5N1)
A/duck/Hunan/S4220/2011(H5N1)
A/Sichuan/26221/2014(H5N6)
A/chicken/Sichuan/NCJPL1/2014(H5N6)
A/duck/Sichuan/NCJPL7/2014(H5N6)
A/cat/Sichuan/SC18/2014(H5N6)
A/environment/Zhenjiang/C13/2013(H5N6)
A/Anas_crecca/Hubei/Chenhu1623-5/2014(H5N6)
A/black_chicken/Jiangxi/10129/2014(H5N6)
A/duck/Jiangxi/13469/2014(H5N6)
A/muscovy_duck/Quang_Ninh/4c111/2013(H5N6)
A/swan_goose/Jilin/JL01/2014(H5N6)
A/goose/Zhejiang/925108/2014(H5N6)
A/goose/Zhejiang/925105/2014(H5N6)
A/duck/Vietnam/LBM360c1-4-1/2013(H5N6)
A/Hubei/1/2010(H5N1)
A/Guangxi/1/2009(H5N1)
A/broiler_duck/Korea/Buan2/2014(H5N8)
A/duck/Hebei/2/2011(H5N2)
A/duck/Eastern_China/1111/2011(H5N2)
A/duck/Jiangsu/m234/2012(H5N2)
A/wild_duck/Shandong/628/2011(H5N1)
A/duck/Jiangsu/k1203/2010(H5N8)
A/goose/Zhejiang/925106/2014(H5N6)
A/duck/Guangdong/wy19/2008(H5N5)
A/duck/Guangdong/wy24/2008(H5N5)
A/Anhui/2/2005(H5N1)
A/goose/Guangdong/1/1996(H5N1)
A/Environment/Shenzhen/1/2015(H5N6)
A/Environment/Shenzhen/2/2015(H5N6)
A/Shenzhen/1/2016(H5N6)
A/Shenzhen/1/2015(H5N6)
A/chicken/Jiangxi/18513/2014(H7N9)
A/chicken/Dongguan/1674/2014(H9N2)
A/Environment/Jiangxi/10615/2014(H10N8)
A/Yunnan/0127/2015(H5N6)
A/environment/Jiangxi/02899/2012(H9N2)
A/environment/Jiangxi/02895/2012(H9N2)
A/environment/Jiangxi/02899/2012(H9N2)
A/CHICKEN/JIANGXI/10963/2014(H7N9)
A/chicken/Rizhao/651/2013(H9N2)
A/Chicken/Nantong/031-2/2013(H9N2)
A/Chicken/Nanjing/023/2013(H9N2)
A/environment/Jiangxi/00452/2013(H9N2)
A/environment/Chongqing/00516/2013(H9N2)
A/chicken/Taixing/10/2010(H9N2)
A/Chicken/Guangdong/SW153/2015(H7N9)
A/chicken/Jiangxi/13521/2014(H7N9)
A/Chicken/Guangdong/SW154/2015(H7N9)
A/pigeon/Zhejiang/2P5/2013(H9N2)
A/chicken/Qingdao/009/2014(H9N2)
A/Environment/Jiangxi/10663/2014(H9N2)
A/chicken/Zhejiang/C1/2013(H9N2)
A/chicken/Huzhou/3861/2013(H9N2)
A/Chicken/Jilin/13188/2014(H7N9)
A/Zhejiang/LS01/2014(H7N9)
A/silkie_chicken/Huzhou/4218/2013(H9N2)
A/environment/Shanghai/S1439/2013(H7N9)
A/chicken/Jiangsu/DTNSZ/2011(H9N2)
A/chicken/Dongguan/3964/2013(H9N2)
A/chicken/Shenzhen/1488/2013(H9N2)
A/chicken/Shenzhen/1047/2013(H9N2)
A/chicken/Shenzhen/515/2013(H9N2)
A/swine/Henan/Y1/2009(H9N2)
A/chicken/Zhejiang/HJ/2007(H9N2)
0.01

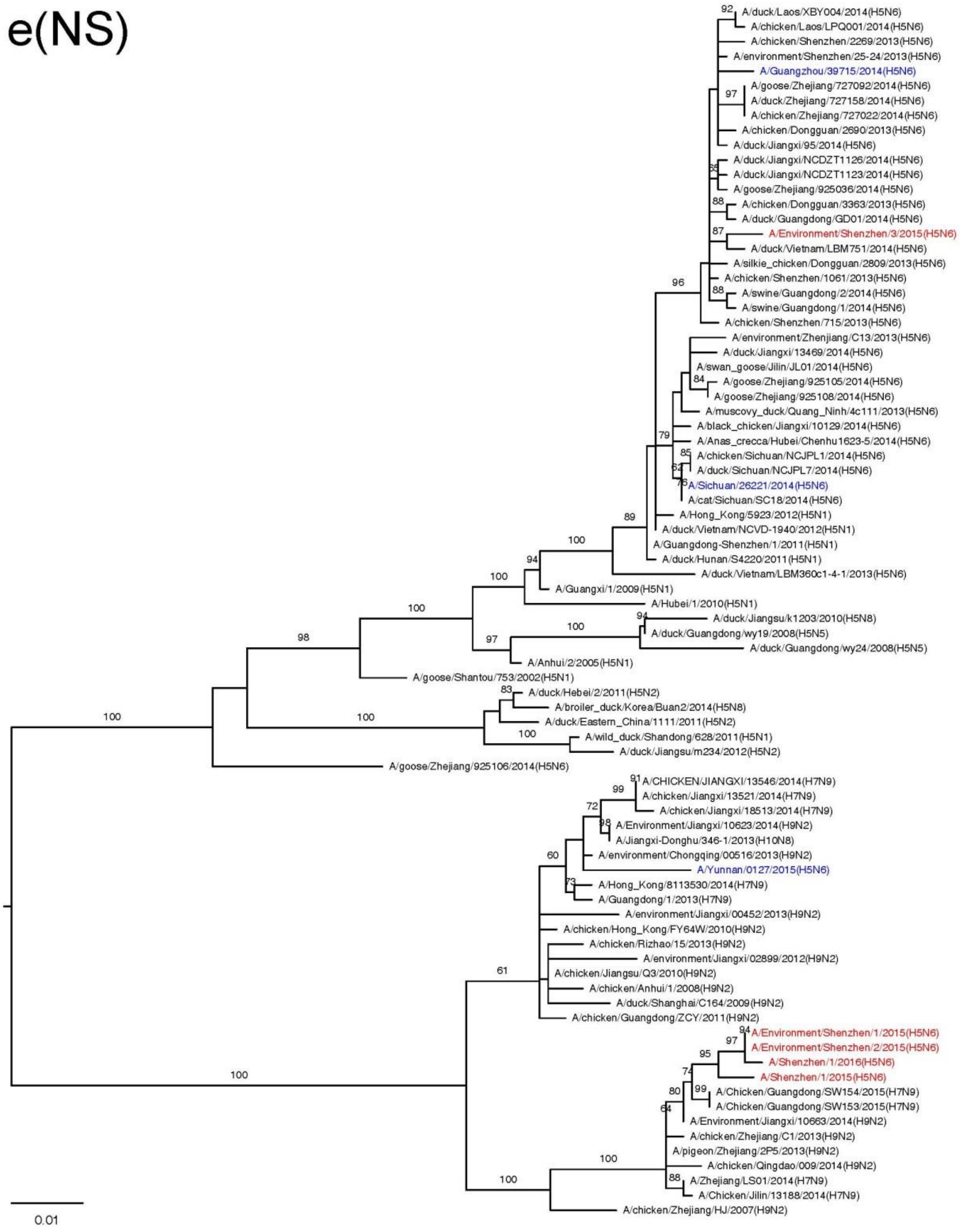

Supplementary Figure S1 Phylogenic trees of PB1, PA, NP, MP and NS genes

The virus name in blue represent previously reported human the H5N6 viruses, and those in red represent the H5N6 identified in this study. **a.**The phylogenic tree of PB1 gene; **b.** The phylogenic tree of PA gene; **c.** The phylogenic tree of NP gene; **d.** The phylogenic tree of MP gene; **e.** The phylogenic tree of NS gene. The bootstrap value of ≥60% were shown.
