# Supplementary material for: Sustained live poultry market surveillance contributes to early warnings for human infection with avian influenza viruses
Source: Emerg Microbes Infect. 2016 Aug 3;5(8):e79–. doi: 10.1038/emi.2016.75 (PMC5034097; doi:10.1038/emi.2016.75)
Supplement: Supplementary Table 1 [file emi201675x2.pdf]

Supplementary Table S1 The accession numbers of the viral sequences isolated in this study

Isolate_Id	PB2 Segment_Id	PB1 Segment_Id	PA Segment_Id	HA Segment_Id	NP Segment_Id	NA Segment_Id	MP Segment_Id	NS Segment_Id	Isolate_Name	Subtype	Passage	Host
EPI_ISL_205313	EPI684538	EPI684539	EPI684537	EPI684541	EPI684534	EPI684540	EPI684536	EPI684535	A/Shenzhen/1/2015	A / H5N6	Original	Human
EPI_ISL_205314	EPI684546	EPI684547	EPI684545	EPI684549	EPI684542	EPI684548	EPI684544	EPI684543	A/Environment/Shenzhen/1/2015	A / H5N6	E1	Environment
EPI_ISL_205315	EPI684554	EPI684555	EPI684553	EPI684557	EPI684550	EPI684556	EPI684552	EPI684551	A/Environment/Shenzhen/2/2015	A / H5N6	E1	Environment
EPI_ISL_205316	EPI684562	EPI684563	EPI684561	EPI684565	EPI684558	EPI684564	EPI684560	EPI684559	A/Environment/Shenzhen/3/2015	A / H5N6	E1	Environment
EPI_ISL_206036	EPI687700	EPI687701	EPI687699	EPI687704	EPI687695	EPI687702	EPI687697	EPI687696	A/Shenzhen/1/2016	A / H5N6	Original	Human
