# Supplementary material for: Sustained live poultry market surveillance contributes to early warnings for human infection with avian influenza viruses
Source: Emerg Microbes Infect. 2016 Aug 3;5(8):e79–. doi: 10.1038/emi.2016.75 (PMC5034097; doi:10.1038/emi.2016.75)
Supplement: Supplementary Table 2 [file emi201675x3.pdf]

Supplementary Table S2 All sequences used from GISAID's EpiFlu database in this study

We acknowledge the authors, originating and submitting laboratories of the sequences from GISAID's EpiFlu™ Database on which this research is based. The list is detailed below. *All submitters of data may be contacted directly via the GISAID website* ***www.gisaid.org***

Segment ID	Segment	Country	Collection date	Isolate name	Originating Lab	Submitting Lab	Authors
EPI515841	MP	China	2013-Apr-16	A/Chicken/Nantong/031-2/2013	Beijing Institute of Microbiology and Epidemiology	Beijing Institute of Microbiology and Epidemiology	Fan, Hang; Liu, Wei; Tong, Yigang; Cao, Wuchun
EPI515833	MP	China	2013-Apr-16	A/Chicken/Nanjing/023/2013	Beijing Institute of Microbiology and Epidemiology	Beijing Institute of Microbiology and Epidemiology	Fan, Hang; Liu, Wei; Tong, Yigang; Cao, Wuchun
EPI530067	NS	China	2013-Dec-02	A/environment/Shenzhen/25-24/2013		BGI Shenzhen	Jinquan,Cheng;Renli,Zhang;Shisong,Fang
EPI530058	NS	China	2014-Jan-10	A/duck/Jiangxi/95/2014		BGI Shenzhen	Bing,Xu;Tao,Zhang;Xiaowen,Li
EPI530061	PA	China	2013-Dec-02	A/environment/Shenzhen/25-24/2013		BGI Shenzhen	Jinquan,Cheng;Renli,Zhang;Shisong,Fang
EPI5300	PA	China	2014-Jan-	A/duck/Jiangxi/95/2014		BGI	Bing,Xu;Tao,Zhang;Xiaowen,Li

53			10			Shenzhen	
EPI530052	PB1	China	2014-Jan-10	A/duck/Jiangxi/95/2014		BGI Shenzhen	Bing,Xu;Tao,Zhang;Xiaowen,Li
EPI530060	PB1	China	2013-Dec-02	A/environment/Shenzhen/25-24/2013		BGI Shenzhen	Jinquan,Cheng;Renli,Zhang;Shisong,Fang
EPI530051	PB2	China	2014-Jan-10	A/duck/Jiangxi/95/2014		BGI Shenzhen	Bing,Xu;Tao,Zhang;Xiaowen,Li
EPI530059	PB2	China	2013-Dec-02	A/environment/Shenzhen/25-24/2013		BGI Shenzhen	Jinquan,Cheng;Renli,Zhang;Shisong,Fang
EPI530063	HA	China	2013-Dec-02	A/environment/Shenzhen/25-24/2013		BGI Shenzhen	Jinquan,Cheng;Renli,Zhang;Shisong,Fang
EPI530054	HA	China	2014-Jan-10	A/duck/Jiangxi/95/2014		BGI Shenzhen	Bing,Xu;Tao,Zhang;Xiaowen,Li
EPI530057	MP	China	2014-Jan-10	A/duck/Jiangxi/95/2014		BGI Shenzhen	Bing,Xu;Tao,Zhang;Xiaowen,Li
EPI530066	MP	China	2013-Dec-02	A/environment/Shenzhen/25-24/2013		BGI Shenzhen	Jinquan,Cheng;Renli,Zhang;Shisong,Fang
EPI530056	NA	China	2014-Jan-10	A/duck/Jiangxi/95/2014		BGI Shenzhen	Bing,Xu;Tao,Zhang;Xiaowen,Li
EPI530065	NA	China	2013-Dec-02	A/environment/Shenzhen/25-24/2013		BGI Shenzhen	Jinquan,Cheng;Renli,Zhang;Shisong,Fang
EPI530064	NP	China	2013-Dec-02	A/environment/Shenzhen/25-24/2013		BGI Shenzhen	Jinquan,Cheng;Renli,Zhang;Shisong,Fang
EPI530055	NP	China	2014-Jan-10	A/duck/Jiangxi/95/2014		BGI Shenzhen	Bing,Xu;Tao,Zhang;Xiaowen,Li
EPI4246	NS	Vietna	2012-Aug-	A/duck/Vietnam/NCVD-1940/201	National	Centers	

66		m	29	2	Centre of Veterinary Diagnostics	for Disease Control and Prevention
EPI424668	PA	Vietnam	2012-Aug-29	A/duck/Vietnam/NCVD-1940/2012	National Centre of Veterinary Diagnostics	Centers for Disease Control and Prevention
EPI424670	PB1	Vietnam	2012-Aug-29	A/duck/Vietnam/NCVD-1940/2012	National Centre of Veterinary Diagnostics	Centers for Disease Control and Prevention
EPI424669	PB2	Vietnam	2012-Aug-29	A/duck/Vietnam/NCVD-1940/2012	National Centre of Veterinary Diagnostics	Centers for Disease Control and Prevention
EPI424672	HA	Vietnam	2012-Aug-29	A/duck/Vietnam/NCVD-1940/2012	National Centre of Veterinary	Centers for Disease

					Diagnostics	Control and Prevention	
EPI284550	HA	Vietnam	2009-Mar-01	A/duck/Vietnam/NCVD-293/2009	National Centre of Veterinary Diagnostics	Centers for Disease Control and Prevention	Davis,Todd; Rivailler,Pierre; Nguyen,Tung
EPI424667	MP	Vietnam	2012-Aug-29	A/duck/Vietnam/NCVD-1940/2012	National Centre of Veterinary Diagnostics	Centers for Disease Control and Prevention	
EPI424665	NP	Vietnam	2012-Aug-29	A/duck/Vietnam/NCVD-1940/2012	National Centre of Veterinary Diagnostics	Centers for Disease Control and Prevention	
EPI466376	PB1	China	2011-Jan-21	A/chicken/Jiangsu/DTNSZ/2011	Yangzhou University	Chinese Academy of Sciences	Daxin, P
EPI466373	MP	China	2011-Jan-21	A/chicken/Jiangsu/DTNSZ/2011	Yangzhou University	Chinese Academy of	Daxin, P

						Sciences	
EPI535188	NP	China	2014-Feb-09	A/Hangzhou/112/2014	Hangzhou Center for Disease Control and Prevention	Hangzhou Center for Disease Control and Prevention	Pu,XY;Li,J;Yu,XF;Kou,Y;Zhou,YY;Qian,X;Pan,JC
EPI457648	MP	China	2013-Apr-03	A/environment/Shanghai/S1439/2013	Harbin Veterinary Research Institute	Harbin Veterinary Research Institute	Zhang, Q.; Shi, J.; Deng, G.; Guo, J.; Zeng, X.; He, X.; Kong, H.; Gu, C.; Li, X.; Liu, J.; Wang, G.; Chen, Y.; Liu, L.; Liang, L.; Li, Y.; Fan, J.; Wang, J.; Li, W.; Guan, L.; Li, Q.; Yang, H.; Chen, P.; Jiang, L.; Guan, Y.; Xin, X.; Jiang, Y.; Tian, G.;
EPI431452	NS	China	2011-Dec-01	A/duck/Hebei/2/2011	Institute of Microbiology, Chinese Academy of Sciences	Institute of Microbiology	Di,Liu; Haigang,Sun; Jinghua,Yan; George F,Gao; Juncai,Ma
EPI431447	PA	China	2011-Dec-01	A/duck/Hebei/2/2011	Institute of Microbiology, Chinese Academy of	Institute of Microbiology	Di,Liu; Haigang,Sun; Jinghua,Yan; George F,Gao; Juncai,Ma

					Sciences		
EPI431446	PB1	China	2011-Dec-01	A/duck/Hebei/2/2011	Institute of Microbiology, Chinese Academy of Sciences	Institute of Microbiology	Di,Liu; Haigang,Sun; Jinghua,Yan; George F,Gao; Juncai,Ma
EPI431445	PB2	China	2011-Dec-01	A/duck/Hebei/2/2011	Institute of Microbiology, Chinese Academy of Sciences	Institute of Microbiology	Di,Liu; Haigang,Sun; Jinghua,Yan; George F,Gao; Juncai,Ma
EPI431448	HA	China	2011-Dec-01	A/duck/Hebei/2/2011	Institute of Microbiology, Chinese Academy of Sciences	Institute of Microbiology	Di,Liu; Haigang,Sun; Jinghua,Yan; George F,Gao; Juncai,Ma
EPI431451	MP	China	2011-Dec-01	A/duck/Hebei/2/2011	Institute of Microbiology, Chinese Academy of Sciences	Institute of Microbiology	Di,Liu; Haigang,Sun; Jinghua,Yan; George F,Gao; Juncai,Ma

EPI431449	NP	China	2011-Dec-01	A/duck/Hebei/2/2011	Institute of Microbiology, Chinese Academy of Sciences	Institute of Microbiology	Di,Liu; Haigang,Sun; Jinghua,Yan; George F,Gao; Juncai,Ma
EPI375506	NS	Hong Kong (SAR)	2012-May-28	A/Hong Kong/5923/2012	Public Health Laboratory Services Branch, Centre for Health Protection	Public Health Laboratory Services Branch, Centre for Health Protection	Mak,G.C.; Cheng,P.K.C.; Lo,J.Y.C.
EPI509882	NS	Hong Kong (SAR)	2014-Mar-17	A/Hong Kong/8113530/2014	Public Health Laboratory Services Branch, Centre for Health Protection	Public Health Laboratory Services Branch, Centre for Health Protection	Mak,G.C.; Cheng,P.K.C.; Lo,J.Y.C.
EPI375503	PA	Hong Kong (SAR)	2012-May-28	A/Hong Kong/5923/2012	Public Health Laboratory Services	Public Health Laboratory Services	Mak,G.C.; Cheng,P.K.C.; Lo,J.Y.C.

					Branch, Centre for Health Protection	Branch, Centre for Health Protection		
EPI375502	PB1	Hong Kong (SAR)	2012-May-28	A/Hong Kong/5923/2012	Public Health Laboratory Services Branch, Centre for Health Protection	Public Health Laboratory Services Branch, Centre for Health Protection		Mak,G.C.; Cheng,P.K.C.; Lo,J.Y.C.
EPI375501	PB2	Hong Kong (SAR)	2012-May-28	A/Hong Kong/5923/2012	Public Health Laboratory Services Branch, Centre for Health Protection	Public Health Laboratory Services Branch, Centre for Health Protection		Mak,G.C.; Cheng,P.K.C.; Lo,J.Y.C.
EPI490879	PB2	Hong Kong (SAR)	2013-Nov-30	A/Hong Kong/5942/2013	Public Health Laboratory Services Branch, Centre for	Public Health Laboratory Services Branch, Centre for		Mak,G.C.; Cheng,P.K.C.; Lo,J.Y.C.

EPI375432	HA	Hong Kong (SAR)	2012-May-28	A/Hong Kong/5923/2012	Health Protection Public Health Laboratory Services Branch, Centre for Health Protection	Health Protection Public Health Laboratory Services Branch, Centre for Health Protection		Mak,G.C.; Cheng,P.K.C.; Lo,J.Y.C.
EPI375434	MP	Hong Kong (SAR)	2012-May-28	A/Hong Kong/5923/2012	Public Health Laboratory Services Branch, Centre for Health Protection	Public Health Laboratory Services Branch, Centre for Health Protection		Mak,G.C.; Cheng,P.K.C.; Lo,J.Y.C.
EPI375504	NP	Hong Kong (SAR)	2012-May-28	A/Hong Kong/5923/2012	Public Health Laboratory Services Branch, Centre for Health Protection	Public Health Laboratory Services Branch, Centre for Health Protection		Mak,G.C.; Cheng,P.K.C.; Lo,J.Y.C.

EPI580391	NS	China	2015-Feb-10	A/Chicken/Guangdong/SW154/2015	South China Agricultural University	South China Agricultural University	Shumin,Xie;Weixin,Jia;Yicun,Lin;Kaixiang,Xing;Xingxing,Ren;Wenbao,Qi;Ming,Liao
EPI580383	NS	China	2015-Feb-05	A/Chicken/Guangdong/SW153/2015	South China Agricultural University	South China Agricultural University	Shumin,Xie;Weixin,Jia;Yicun,Lin;Kaixiang,Xing;Xingxing,Ren;Wenbao,Qi;Ming,Liao
EPI580386	PA	China	2015-Feb-10	A/Chicken/Guangdong/SW154/2015	South China Agricultural University	South China Agricultural University	Shumin,Xie;Weixin,Jia;Yicun,Lin;Kaixiang,Xing;Xingxing,Ren;Wenbao,Qi;Ming,Liao
EPI580378	PA	China	2015-Feb-05	A/Chicken/Guangdong/SW153/2015	South China Agricultural University	South China Agricultural University	Shumin,Xie;Weixin,Jia;Yicun,Lin;Kaixiang,Xing;Xingxing,Ren;Wenbao,Qi;Ming,Liao
EPI580377	PB1	China	2015-Feb-05	A/Chicken/Guangdong/SW153/2015	South China Agricultural University	South China Agricultural University	Shumin,Xie;Weixin,Jia;Yicun,Lin;Kaixiang,Xing;Xingxing,Ren;Wenbao,Qi;Ming,Liao

EPI580385	PB1	China	2015-Feb-10	A/Chicken/Guangdong/SW154/2015	South China Agricultural University	South China Agricultural University	Shumin,Xie;Weixin,Jia;Yicun,Lin;Kaixiang,Xing;Xingxing,Ren;Wenbao,Qi;Ming,Liao
EPI580384	PB2	China	2015-Feb-10	A/Chicken/Guangdong/SW154/2015	South China Agricultural University	South China Agricultural University	Shumin,Xie;Weixin,Jia;Yicun,Lin;Kaixiang,Xing;Xingxing,Ren;Wenbao,Qi;Ming,Liao
EPI580376	PB2	China	2015-Feb-05	A/Chicken/Guangdong/SW153/2015	South China Agricultural University	South China Agricultural University	Shumin,Xie;Weixin,Jia;Yicun,Lin;Kaixiang,Xing;Xingxing,Ren;Wenbao,Qi;Ming,Liao
EPI580382	MP	China	2015-Feb-05	A/Chicken/Guangdong/SW153/2015	South China Agricultural University	South China Agricultural University	Shumin,Xie;Weixin,Jia;Yicun,Lin;Kaixiang,Xing;Xingxing,Ren;Wenbao,Qi;Ming,Liao
EPI580390	MP	China	2015-Feb-10	A/Chicken/Guangdong/SW154/2015	South China Agricultural University	South China Agricultural University	Shumin,Xie;Weixin,Jia;Yicun,Lin;Kaixiang,Xing;Xingxing,Ren;Wenbao,Qi;Ming,Liao

EPI580388	NP	China	2015-Feb-10	A/Chicken/Guangdong/SW154/2015	South China Agricultural University	South China Agricultural University	Shumin,Xie;Weixin,Jia;Yicun,Lin;Kaixiang,Xing;Xingxing,Ren;Wenbao,Qi;Ming,Liao
EPI580380	NP	China	2015-Feb-05	A/Chicken/Guangdong/SW153/2015	South China Agricultural University	South China Agricultural University	Shumin,Xie;Weixin,Jia;Yicun,Lin;Kaixiang,Xing;Xingxing,Ren;Wenbao,Qi;Ming,Liao
EPI541773	PA	Taiwan	2013-Dec-27	A/Taiwan/3/2013	Centers for Disease Control	Taiwan CDC	
