# Supplementary material for: Sustained live poultry market surveillance contributes to early warnings for human infection with avian influenza viruses
Source: Emerg Microbes Infect. 2016 Aug 3;5(8):e79–. doi: 10.1038/emi.2016.75 (PMC5034097; doi:10.1038/emi.2016.75)
Supplement: Supplementary Table 3 [file emi201675x4.pdf]

Supplementary Table S3 RT-PCR result of environmental samples collected from intensive LPMs surveillance in ten districts in Shenzhen

District	June-November, 2015				December, 2015							January, 2016						
	No.of Sample	No.of positive sample		Positive rate of H9N2 (%)	No.of Sample	No.of positive sample				Positive rate of H5N6 (%)*	Positive rate of H9N2 (%)*	No.of Sample	No. of positive sample				Positive rate of H5N6 (%)*	Positive rate of H9N2 (%)*
		FluA	H9N2			FluA	H5N6	H9N2	H5N6/ H9N2				FluA	H5N6	H9N2	H5N6/ H9N2		
BA	0	0	0	0	20	14	3	4	2	25	30	10	0	0	0	0	0	0
DP	0	0	0	0	20	14	3	3	3	30	30	10	8	0	8	0	0	80
FT	0	0	0	0	20	16	2	1	11	65	60	10	10	0	10	0	0	100
GM	0	0	0	0	20	17	5	3	6	55	45	10	7	0	3	0	0	30
LG	60	34	15	25	20	8	2	0	1	15	5	10	4	0	0	3	30	30
LH	0	0	0	0	20	12	0	6	1	5	35	10	8	0	2	0	0	20
LHX	0	0	0	0	20	16	7	0	4	55	20	10	10	0	5	0	0	50
NS	60	0	0	0	20	18	0	15	0	0	75	20	19	0	6	10	50	80
PS	0	0	0	0	20	15	2	2	6	40	40	10	10	1	3	3	40	60
YT	0	0	0	0	20	10	0	1	0	0	5	10	10	1	2	6	70	80
**Total**	120	34	15	25 (95%CI 20%-30%)	200	140	24	35	34	29 (95%CI 14%-44%)	34.5 (95%CI 21%-48%)	110	86	2	39	22	19 (95%CI 3%-35%)	53 (95%CI 33%73%)

Footnote: As H7N9, H5N1 or H10N8 were not detected in this study, this table showed the results of H9N2 and H5N6 virus detected in this study. *: The positive rate for each subtype included the mixed samples both positive for H9N2 or H5N6.
