# Supplementary material for: Sustained live poultry market surveillance contributes to early warnings for human infection with avian influenza viruses
Source: Emerg Microbes Infect. 2016 Aug 3;5(8):e79–. doi: 10.1038/emi.2016.75 (PMC5034097; doi:10.1038/emi.2016.75)
Supplement: Supplementary Table 4 [file emi201675x5.pdf]

**Supplementary Table S4 RT-PCR result of environmental samples collected from epidemiologic linked LPMs of the patients**

| Site | Sample type | No. of samples | No. of positive sample for subtype H5 and N6 | Positive rate (%) |
|---|---|---|---|---|
| Epidemiological linked LPMs of patient 1 | Poultry feces | 9 | 0 | 0 |
| | Drinking water | 1 | 0 | 0 |
| | Chicken feather | 1 | 1 | 100 |
| | Sewage | 5 | 2 | 40 |
| | Swabs from processing tools and feeders | 12 | 4 | 33 |
| Epidemiological linked LPMs of patient 2 | Poultry feces | 7 | 4 | 57 |
| | Drinking water | 1 | 0 | 0 |
| | Sewage | 2 | 0 | 0 |
| | Cage swabs | 5 | 1 | 20 |
| | Swabs from poultry meat | 4 | 0 | 0 |
| | Swabs from processing tools, floor and table | 33 | 6 | 18 |
| Total | | 80 | 18 | 23 |
